# Gastrointestinal Parasite Community and Phenotypic Plasticity in Native and Introduced Alien Lagomorpha

**DOI:** 10.3390/ani12101287

**Published:** 2022-05-17

**Authors:** Stefano Catalano, Valentina La Morgia, Anna Rita Molinar Min, Angela Fanelli, Pier Giuseppe Meneguz, Paolo Tizzani

**Affiliations:** 1 Department of Veterinary Medicine and Animal Production, University of Naples Federico II, 80137 Naples, Italy; ste.cata85@gmail.com; 2 Moredun Research Institute, Pentlands Science Park, Penicuik EH26 0PZ, UK; 3 Institute for Environmental Protection and Research (ISPRA), Via Ca’ Fornacetta 9, Ozzano Emilia, 40064 Bologna, Italy; valentina.lamorgia@isprambiente.it; 4 Department of Veterinary Sciences, University of Turin, Largo Paolo Braccini 2, Grugliasco, 10095 Turin, Italy; annarita.molinar@unito.it (A.R.M.M.); piergiuseppe.meneguz@unito.it (P.G.M.); 5 Department of Veterinary Medicine, University of Bari, Valenzano, 70010 Bari, Italy; angela.fanelli@uniba.it

**Keywords:** Lagomorpha, parasite diversity, parasite-mediated competition, invasive species, Lepus, Sylvilagus

## Abstract

**Simple Summary:**

The introduction of alien species outside their native range represents one of the main threats to biodiversity conservation and it is mainly driven by human activities. In this work, we used the introduction of an alien lagomorph (*Sylvilagus floridanus*) outside its natural range as a case study to describe and evaluate the long-term effects on the native lagomorph community. In particular, *S. floridanus* was host to different exotic parasites, which adapted to new biotic and abiotic conditions and, as a consequence, invaded and largely altered the parasite community of native Lagomorpha species. This work represents the first exploratory study on the ecological macro-community formed by native and alien host species and their parasitofauna. Further efforts are needed to fully understand the strategies and factors conditioning the evolution of these specific parasite communities, and more in general the factors influencing the effect and evolution of alien pathogens in a new geographic area.

**Abstract:**

The Convention on Biological Diversity classifies “Invasive Alien Species” as those whose introduction and spread represents a threat for biodiversity. Introduction of alien pathogens, including parasites, is one of the main consequences of the introduction of invasive alien species. The objective of this work was to assess the parasite community composition in native lagomorphs *(Lepus europaeus* and *Lepus timidus varronis*) in sympatric and non-sympatric conditions with an alien lagomorph (*Sylvilagus floridanus*), and to evaluate the phenotypic traits of exotic parasites in such conditions. We firstly describe the characteristics of the parasite community in the different host species (richness, prevalence, abundance and intensity), and, secondly, the phenotypic traits of the observed parasite species in each host. Nine helminths were reported on: eight nematodes (*Obeliscoides cuniculi*, *Trichostrongylus calcaratus*, *Trichostrongylus retortaeformis*, *Trichostrongylus affinis*, *Trichuris leporis*, *Trichostrongylus colubriformis*, *Passalurus ambiguus*, and *Nematodirus* sp.) and one unidentified cestode. In addition, exotic parasites showed significantly different phenotypic plasticity after spillover from *S. floridanus* to *L. europaeus*, whereas endemic parasite species were not isolated in the alien *S. floridanus.* Our results highlight that the community of autochthonous and allochthonous Lagomorpha in northwestern Italy represents an extremely interesting system for modelling ecological and evolutionary interactions between parasites and their hosts.

## 1. Introduction

The anthropogenic impact on many ecosystems across the globe includes the movement of live organisms, which represents one of the main threats to local biodiversity. The species whose introduction and/or spread represents a threat for biodiversity through their impact on ecosystems, habitats or species, are classified as “Invasive Alien Species” by the Convention on Biological Diversity [1,2]. Over time, humans have often introduced animal and plant species outside their natural range. In some cases, introductions have been unintentional, as in the case of species that have been accidentally transported through the movement of commercial goods (e.g., as stowaways in containers), which probably happened for the brown rats (*Rattus norvegicus* Berkenhout, 1769) [3,4]. Farms, zoos, aquariums and botanical gardens have in the past played a role in this context, introducing species that have escaped from controlled situations and, in some cases, have been able to adapt, settle, and eventually expand in their new ecosystem. The African sacred ibis (*Threskiornis aethiopicus* Latham, 1790) has escaped from zoological parks in many countries [5], and a population of Siberian chipmunks (*Eutamias sibiricus* Laxmann, 1769) in the Netherlands originated from a group of animals left behind after the removal of a zoo [6]. Several species also escaped from fur farms, e.g., the American mink (*Neovison vison* Schreber, 1777) and the coypu (*Myocastor coypus* Molina, 1782) [7]. Recently, the pet trade has been an important source of exotic animal introductions to Europe. The common slider (*Trachemys scripta*) is a very popular reptile worldwide, and often its introductions resulted from the escape or release of pets [7]. Similarly, the rose-ringed parakeet (*Psittacula krameri* Scopoli, 1769) populations mostly originated from escapes from private collections [8]. Other introductions have been intentional, as in the case of species introduced for fishing (the common carp *Cyprinus carpio*), hunting (various mammals belonging to the orders Artiodactyla and Lagomorpha, as birds within the Galliformes and Anseriformes), and/or biological control (the arlequin ladybird *Harmonia axyridis* (Pallas, 1773) released for the biocontrol of aphids) [9].

Some of these species have been responsible for negative impacts on natural ecosystems and have been recognised as a major threat to biodiversity conservation. Whenever an alien species is introduced and established into a new area, it can exert a negative impact on the environment and ecosystems, representing a threat to native species, with which it could compete directly or indirectly for resources [10,11] or with which it could act as a predator [12], thus altering the local food chain. An introduced species could also cause habitat modifications and hybridise with related species or varieties [13,14]. In Europe, regulations on the introduction of wildlife are stringent and take into account various international provisions, from the Washington Convention, the Convention on Biological Diversity and various codes of conduct, as well as the EU provisions of the Habitats (92/43/EEC) and Birds (79/409/EEC) Directives and CITES Regulation 338/97/EEC, up to the most recent Regulation (EU) No 1143/2014 on invasive alien species. These provisions are then declined by Member States at a national level, considering the key principle that the translocation of a species should not pose a risk to the conservation of the ecosystem and its components. Each translocation should therefore be accompanied by a careful risk analysis, carried out with a holistic and interdisciplinary approach since it is not only the single individual that is introduced but its related pathogenic agents too, which include viruses, bacteria, parasites and fungi [15].

As a consequence, when a species is introduced into a new area, specific risks such as virus or parasite introduction must be taken into account. Infectious agents introduced with allochthonous species can influence local dynamics, especially when the introduced host acts as a reservoir from which the infection can spill over to native species. Hosts and parasites are intimately linked by trophic interactions that affect the ecological community and are also responsible for reducing host fitness, both at the population and individual level [16]. Several studies [17,18,19,20,21,22,23] have stressed the importance of the role of parasites in destabilizing host dynamics. Other dynamics of invasions have been relatively less explored; for instance, introduced species may act as a reservoir and amplifiers for parasites hosted in autochthonous species, from which infections could then spill back to native fauna [24,25].

The effects and consequences of virus introduction have been better studied than for parasites, with a significant gap in the effect on sympatric mammal communities. In addition, the approach to parasitological studies in wildlife is often limited to the description of epidemiological indicators (prevalence, intensity and abundance of infection/infestation), without exploring the relationship between parasites and their hosts. There is therefore a lack of studies on the complex relationships between host, parasite and environment. Moreover, studies often focus on the host species, without an assessment of parasite communities and parasitic adaptations in new biotic and abiotic environments.

The spillover of alien parasites into autochthonous host populations unfolded after the repeated introduction of the eastern cottontail rabbit (*Sylvilagus floridanus* J.A. Allen, 1890) for hunting purposes in several regions of Europe [26,27,28], and it has been further documented by relatively recent studies [29,30].

In northwestern Italy, *L. europaeus* is a common species. In the province of Alessandria, its densities can be influenced by restocking activities, as well as environmental conditions [31], and they can reach up to 50 hares/ha in protected lowland areas along floodplains [32,33,34]. Here, the species is sympatric with *S. floridanus*, with a total range overlap between the two species.

The eastern cottontail rabbit was introduced in Italy in the 1960s [26], and its population has recorded an enormous increase in the province of Alessandria since the 1980s, with an expansion that continued until 1996–1997. Since 1998, the area of presence seems to have stabilized, settling at around 620–660 km^2^ [35]. Currently, *S. floridanus* local densities vary between 10 and 100 individuals/km^2^ [36].

In the province of Cuneo, and more specifically in its Varaita Valley (Latitude: 44.57963, Longitude: 7.18452), *L. europaeus* has a range of about 31,000 ha with an estimated density of 2–4 hares/100 ha [37]. The range of *L. t. varronis* expands over about 16,000 ha at higher elevation, with densities of 1–3 hares/100 ha; this distribution partially overlaps with *L. europaeus* in the lower part of its range [37]. The population of *S. floridanus* is relegated to the lower valley, and it only partially overlaps with *L. europaeus*, with densities of 5–10 animals/100 ha [37]. Therefore, in this study area, the three host species have a partially overlapping range: *L. europaeus* is partially sympatric with both *L. t. varronis* and *S. floridanus*, while populations of *L. t. varronis* and *S. floridanus* are totally separated.

The study of the gastrointestinal parasitofauna within the digestive tract of the lagomorph community in northwestern Italy specifically enables us to both describe parasite community richness and evaluate the species-specificity and morpho-functional adaptations of alien parasitic species within three lagomorph hosts (i.e., the introduced *S. floridanus*, and the autochthonous brown hare (*Lepus europaeus* Pallas, 1778) and mountain hare (*Lepus timidus varronis* Angermann, 1967)).

The specific objective of this work was to assess the parasite community composition in native lagomorphs, in sympatric and non-sympatric conditions with *S. floridanus*, and to evaluate the phenotypic traits of exotic parasites in such conditions. We focused on quantitative measures of individual parasites and their morphological structures that may be indicators of fitness within the parasitised host species. Intra-specific variation in the biometric measurements of the observed parasites are then proposed to be an effective indicator to measure the life history traits of a parasite species and its adaptation to its host.

## 2. Materials and Methods

### 2.1. Study Area

**Figure 1:**
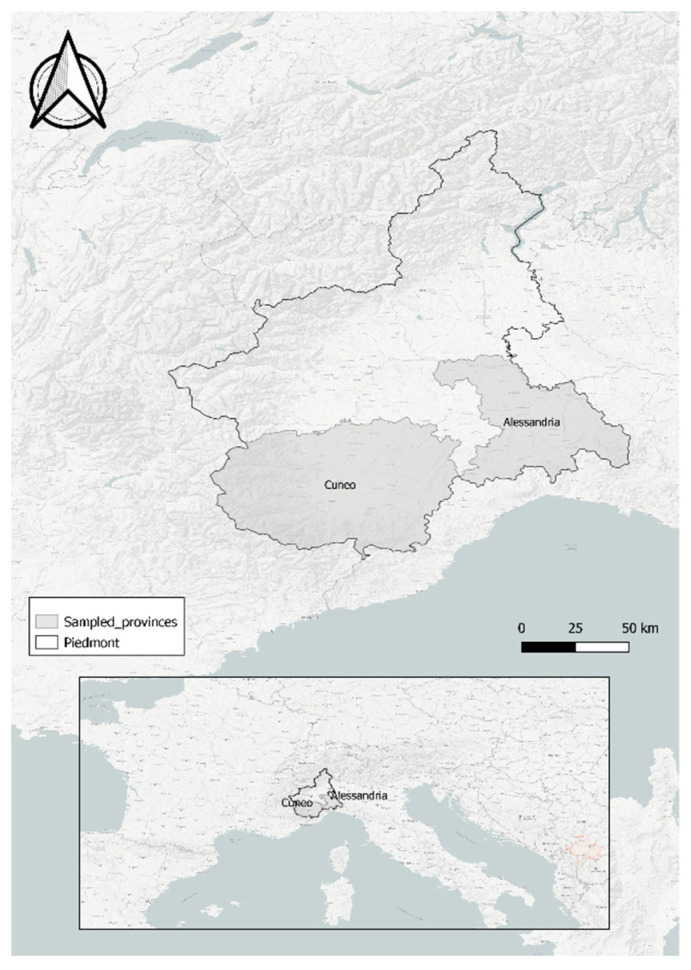
Piedmont region and localization of the sampled provinces of Alessandria and Cuneo in northwestern Italy.

The research was conducted in two different provinces (Alessandria and Cuneo) of the Piedmont Region in northwestern Italy (Figure 1). We sampled three species within the order Lagomorpha: the autochthonous brown hare and mountain hare, and the alien eastern cottontail rabbit.

### 2.2. Collection of Specimens and Parasitological Analysis

Gastrointestinal tracts were obtained at the post-mortem examination of individuals harvested during the hunting season (i.e., *L. europaeus* and *L. t. varronis*) or, for *S. floridanus*, in the context of a plan aimed at the numerical control of the species. According to the Italian regulation no. 157/92, local agencies approve hunting bags and/or implement control activities based on harvest plans reviewed by Institute for Environmental Protection and Research. Population control operations of alien species are also in accordance with Article 29 of Piedmont Regional Law no. 70/96. Gastrointestinal tracts were collected as soon as possible after the animal’s death and were stored at −20 °C.

Samples of *L. europaeus* were collected during the hunting seasons from 2003/2004 to 2005/2006 (from September to December) for the province of Cuneo, whereas they were collected only during the 2003/2004 (from September to December) hunting season for the province of Alessandria. Samples of *S. floridanus* were collected during the hunting seasons from 2004/2005 to 2008/2009 (from September to December) for the province of Alessandria. Samples of *L. t. varronis* were collected during the hunting seasons from 2004/2005 to 2007/2008 (from September to December) for the province of Cuneo.

Overall, the survey of the gastrointestinal parasite fauna of lagomorphs was carried out on 74 individuals; 30 *L. europeaeus* and 7 *L. t. varronis* from the alpine environment (i.e., Varaita Valley in the Cuneo Province), and 21 *L. europaeus* and 16 *S. floridanus* from the province of Alessandria.

After thawing, each portion of the gastrointestinal tract (i.e., stomach, small intestine, caecum and large intestine) was longitudinally dissected, washed using tap water, and sieved (mesh size 75 μm). The filtrate was diluted with tap water and observed under a Nikon stereoscope (model SMZ-2T) to search for and collect any parasites present. All nematodes found in the gastrointestinal tract were counted in order to determine the infection intensity, then they were identified by using morphological identification keys [38] before preservation in 70% ethyl alcohol.

### 2.3. Biometric Measurements of Parasites

Biometric measurements of spicula and gubernaculum (reproductive organs in male parasites within the phylum Nematoda) were carried out only on the alien parasite species shared between *S. floridanus* and *L. europaeus* to evaluate the phenotypic plasticity of exotic parasites in alien and native hosts. In addition to the biometric measurements in male parasites, fertility in female parasites was assessed by counting the number of eggs contained within the uterus. This measurement was taken only for the strongylids *Trichostrongylus calcaratus* (Ransom, 1911) and *Trichostrongylus affinis* (Graybill, 1924) since it was not possible to accurately evaluate this indicator in the gastric nematode *Obeliscoides cuniculi* (Graybill, 1923).

In order to obtain accurate biometric measurements, the reproductive apparatus of all male parasites was photographed through the eyepiece equipped with the micrometer scale of a Nikon optical microscope (model Eclipse E400) at a magnification of 100–400× and using a Panasonic DMC-TZ4 camera.

### 2.4. Software for Image Analysis

In order to precisely analyse biometric measurements, which are traditionally collected using an optical microscope equipped with a micrometric scale, the images of the male reproductive apparatus (spicula and gubernaculum where present), were imported into a geographic information system (GIS) using QGIS 3.10 software [39]. This software, normally used for the measurement of geographically referenced objects, was in this case adapted to improve the morphological analysis of the observed anatomical structures. The application of GIS software for collecting biometric measurements of nematodes was previously described [40]. The biometric measures of spicula and gubernaculum taken with this approach, have to be considered as planar areas to give an approximation of the real size of the anatomic structures. When speaking about “size” in the rest of the manuscript, this approximation has to be considered to better interpret the results.

### 2.5. Statistical Analysis

The parasite community has been described using the common epidemiological indicators of prevalence (proportion of infected hosts), abundance (average number of parasites calculated on the total number of analysed individual hosts) and intensity (average number of parasites calculated on the total number of positive individual hosts), according to Margolis [41] and Bush [42].

The differences among biometric measures taken in different hosts were evaluated using the Mann–Whitney test. Statistical significance was considered for a *p*-value < 0.05. All the analyses were carried out with R software 4.1.1 [43]. Graphs were created in R by using the package “ggpubrr” [44] and “VennDiagram” [45].

## 3. Results

### 3.1. Description of the Parasite Community

**Figure 2:**
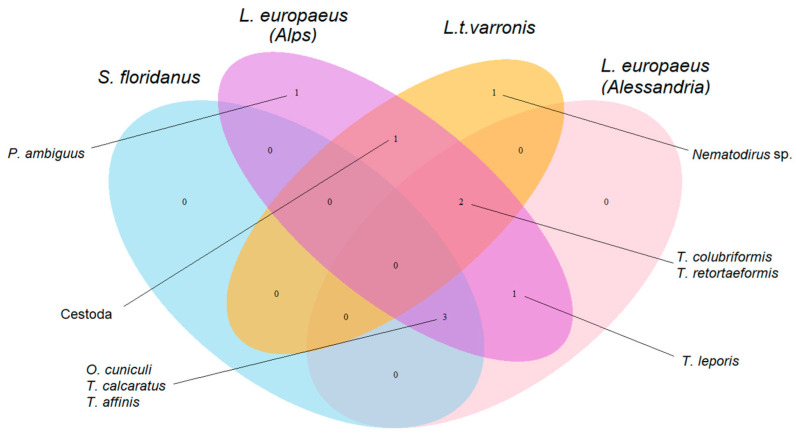
Description of the parasite community reported in the different lagomorph hosts (i.e., *Lepus europaeus*, *Lepus timidus varronis* and *Sylvilagus floridanus*) from the provinces of Alessandria and Cuneo (herein named Alps) in northwestern Italy.

The parasitological analysis carried out on the lagomorphs revealed the presence of nine helminths: eight nematodes (*O. cuniculi*, *T. calcaratus*, *Trichostrongylus retortaeformis* Zeder, 1800, *T. affinis*, *Trichuris leporis* Frölich, 1789, *Trichostrongylus colubriformis* Giles, 1892, *Passalurus ambiguus* Rudolphi, 1819, and *Nematodirus* sp. Molineidae Skrjabin & Schulz, 1937) and one unidentified cestode. The observed parasite species, grouped by host and sampling locality, are summarised in Figure 2.

The component parasite community (see definition by Bush [42]) had variable richness depending on the host, with *L. europaeus* showing the highest richness and *S. floridanus* the lowest one. Out of the nine parasite species that we found, eight (88.8%) were isolated from the gastrointestinal tract of *L. europaeus* of the Cuneo Province, six (66.6%) from *L. europaeus* of the Alessandria Province, five (55.5%) from *L. t. varronis* of the Cuneo Province, and three (33.3%) from *S. floridanus* of the Alessandria Province.

The characteristics of the parasite community, in terms of prevalence, abundance, and intensity, along with their 95% confidence interval (CI) are shown in Table 1.

### 3.2. Biometric Measurements of Parasites

**Figure 3:**
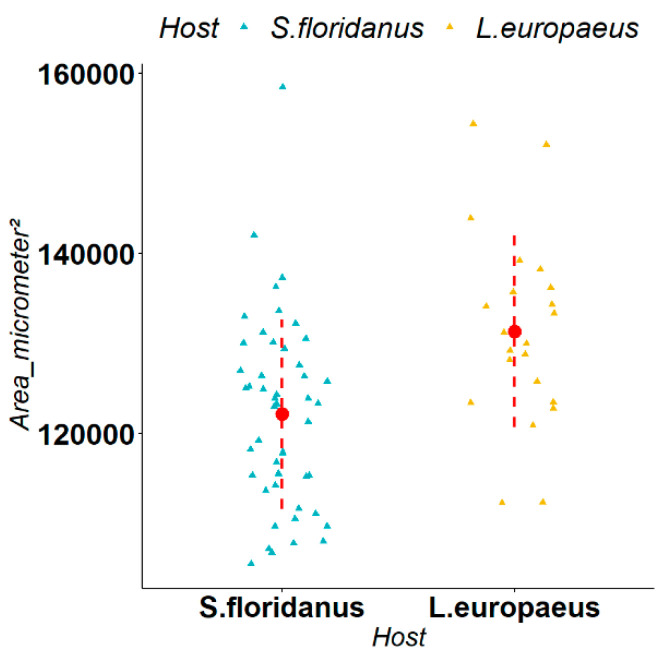
Comparison of the spicula of *Obeliscoides cuniculi* in *Lepus europaeus* and *Sylvilagus floridanus* (measurements shown in μm^2^). The triangles represent the single observations, the red dots represent the average values, while the red dashed lines represent the standard deviation.

*Obeliscoides cuniculi* is a parasite typical of the stomach of *S. floridanus* in its native range and, during our study, it was isolated in both *S. floridanus* and *L. europaeus*. We observed that the average size of its spicula in *L. europaeus* and *S. floridanus* was respectively 131,347 μm^2^ and 122,138 μm^2^. The Mann–Whitney U-test indicated a statistically significant difference (W = 783, *p*-value = 0.001) in the size of the spicula in the two different host species (Figure 3).

**Figure 4:**
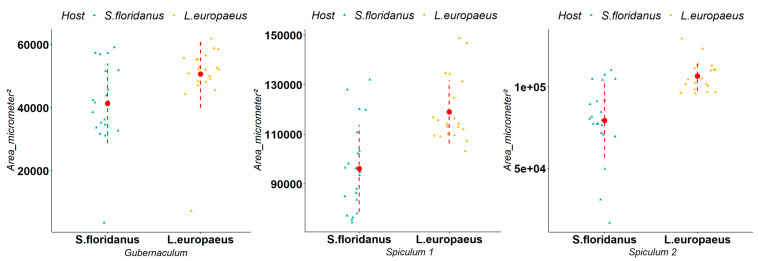
Comparison of the gubernaculum, spiculum 1 and spiculum 2 of *Trichostrongylus calcaratus* in *Lepus europaeus* and *Sylvilagus floridanus* (measurements shown in μm^2^). The triangles represent the single observations, the red dots represent the average values, while the red dashed lines represent the standard deviation.

**Figure 5:**
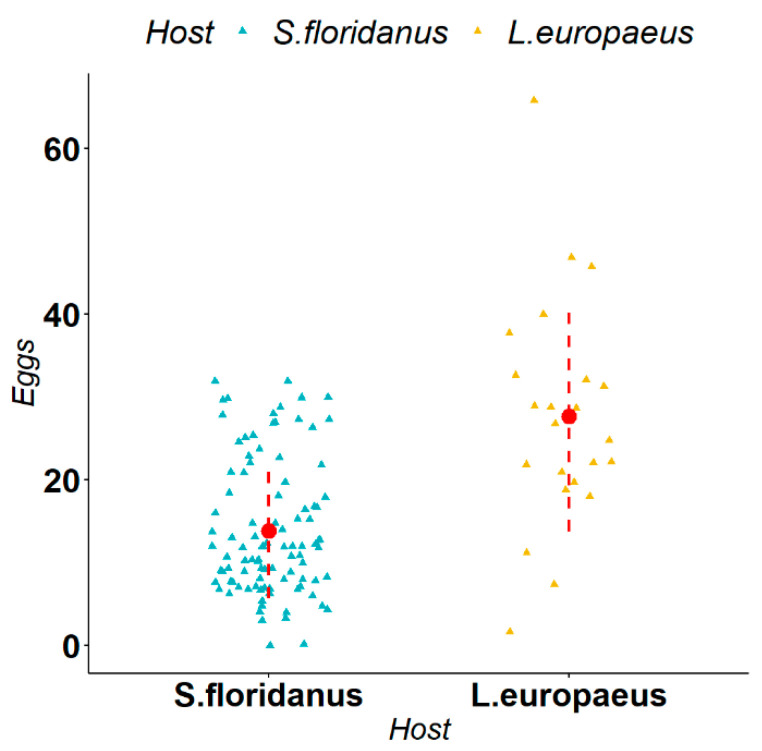
Comparison of female fertility (i.e., in utero count of the number of eggs) of *Trichostrongylus calcaratus* in *Lepus europaeus* and *Sylvilagus floridanus*. The triangles represent the single observations, the red dots represent the average values, while the red dashed lines represent the standard deviation.

*Trichostrongylus calcaratus* is a parasite typical of the small intestine of *S. floridanus* in its native range and, during our study, it was isolated in both *S. floridanus* and *L. europaeus*. We observed that the average size of measurements in *L. europaeus* and *S. floridanus* were respectively 50,700 μm^2^ and 41,350 μm^2^ for the gubernaculum, 119,906 μm^2^ and 96,261 μm^2^ for spiculum 1, and 106,570 μm^2^ and 79,508 μm^2^ for spiculum 2. The Mann–Whitney U-test indicated a statistically significant difference (respectively Gubernaculum (W = 354, *p*-value = 0.002), spiculum 1 (W = 371, *p*-value < 0.001), spiculum 2 (W = 407, *p*-value < 0.001)) in the size of all the measurements in the two different host species (Figure 4). Regarding female fertility, we observed that the average number of eggs in *L. europaeus* and *S. floridanus* was respectively 28 and 14 eggs. The Mann–Whitney U-test indicated a statistically significant difference (W = 1835, *p*-value < 0.001) in the in utero egg counts in the two different host species (Figure 5).

**Figure 6:**
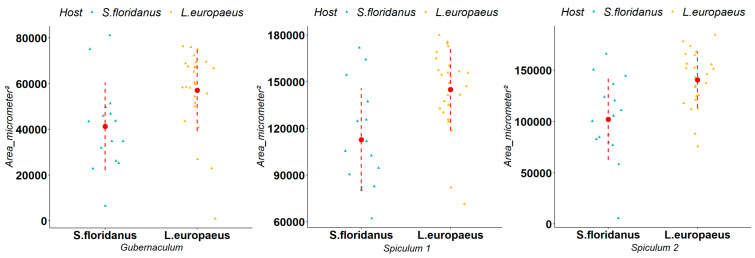
Comparison of the gubernaculum, spiculum 1 and spiculum 2 of *Trichostrongylus affinis* in *Lepus europaeus* and *Sylvilagus floridanus* (measurements shown in μm^2^). The triangles represent the single observations, the red dots represent the average values, while the red dashed lines represent the standard deviation.

**Figure 7:**
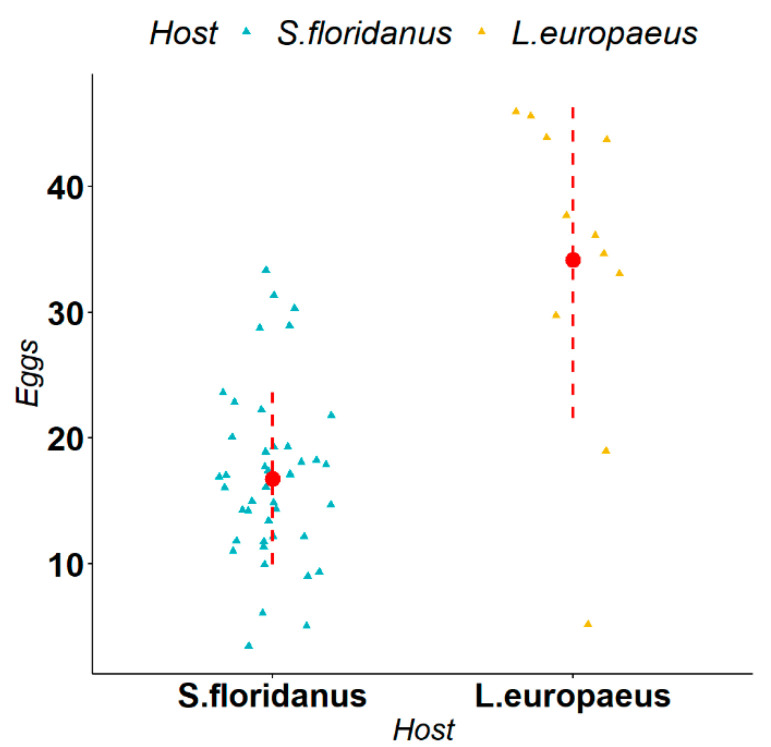
Comparison of female fertility (i.e., in utero count of the number of eggs) of *Trichostrongylus affinis* in *Lepus europaeus* and *Sylvilagus floridanus*. The triangles represent the single observations, the red dots represent the average values, while the red dashed lines represent the standard deviation.

*Trichostrongylus affinis* is a parasite typical of the large intestine of *S. floridanus* in its native range and, during our study, it was isolated in both *S. floridanus* and *L. europaeus*. We observed that the average size of measurements in *L. europaeus* and *S. floridanus* were respectively 50,049 μm^2^ and 41,293 μm^2^ for gubernaculum, 144,905 μm^2^ and 112,734 μm^2^ for spiculum 1, and 140,575 μm^2^ and 102,110 μm^2^ for spiculum 2. The Mann–Whitney U-test indicated a statistically significant difference respectively Gubernaculum (W = 295, *p*-value = 0.006), spiculum 1 (W = 302, *p*-value = 0.003), spiculum 2 (W = 307, *p*-value = 0.001) in the size of all the measurements in the two different host species (Figure 6). Regarding female fertility, we observed that the average number of eggs in *L. europaeus* and *S. floridanus* was respectively 34 and 17 eggs. The Mann–Whitney U-test indicated a statistically significant difference (W = 407, *p*-value < 0.001) in the in utero egg counts in the two different host species (Figure 7).

## 4. Discussion

This work represents one of the few studies assessing and comparing the variability of the parasite community across different host species. We evaluated multi-parasitism in a sympatric, multi-host community. Further interest to our research is added by the fact that the comparison included alien and autochthonous lagomorph species characterised by different life history traits, environmental needs and variable degrees of spatial and temporal overlapping. The other focus that makes this investigation worthy of interest is the assessment of both parasite composition at a community level, and phenotypic plasticity at an individual level, to assess the potential adaptation of exotic parasite species shared among native and alien hosts.

Our study highlights clear dynamics in the invasion of the autochthonous parasite community, with alien parasite species infecting the autochthonous *L. europaeus*, whereas the latter do not seem to harbour parasites capable of infecting the alien *S. floridanus*. These results should certainly be viewed with caution, considering that the small sample size may have reduced the possibility of detecting *Lepus* parasites in *S. floridanus*. However, our data clearly document the parasite transmission from the alien lagomorph to native hares. Furthermore, our findings demonstrate that alien parasites, when introduced in an autochthonous population, may have significantly higher life history traits, which may be indicative of increased parasite fitness, as we observed in *L. europaeus*.

Parasite community composition and phenotypic adaptations further contribute to studying the role of pathogens influencing wildlife population dynamics and ecology [46,47,48,49]. Examples of a similar approach are the evaluation of the dynamics of sarcoptic mange in different cervid and bovid species [50], the analysis of the gastrointestinal parasite community in a multi-host ungulate system in southern Spain [51,52], or the assessment of the richness of parasite community in alpine Galliformes [53]. Nevertheless, scarce information is still available for other taxa, including lagomorphs.

The analysis of the three host species enabled us to compile a complete checklist of the parasite community in our study area. Of particular interest, among the parasite species detected, we found that: (I) in *S. floridanus*, we detected only parasite species (*O. cuniculi*, *T. calcaratus* and *T. affinis*) that belong to the common parasitofauna described in this host in its original range (see also Tizzani [29,30]); (II) in *L. europaeus*, we reported three alien parasites (i.e., *O. cuniculi*, *T. calcaratus* and *T. affinis*) transmitted by the alien *S. floridanus* [54]; and (III) in terms of parasite richness, *S. floridanus* was the host species with the least diverse parasite community (three species), followed by *L. t. varronis* (five species) and *L. europaeus* (eight species). All the other parasite species were already reported in previous studies in Italy and Europe [29,54,55,56,57], although some common parasites of hares (e.g., *Graphidium strigosum* Dujardin 1845, *Nematodiroides zembrae* Bernard, 1965) [58] were not detected in our study area. In contrast to the isolation of alien parasite species in *L. europaeus*, the absence of any atypical, endemic parasites in the gastrointestinal tract of *S. floridanus* may reflect previous experience showing that the prevalence of autochthonous parasite species in introduced animal populations can be less than half that in native animal populations [59].

The study of the prevalence, abundance and intensity of parasite communities led to the following conclusions: (I) in *L. europaeus* in the province of Alessandria, the highest values of prevalence, abundance and intensity belonged to the exotic species *O. cuniculi*, *T. calcaratus* and *T. affinis*; (II) in *L. europaeus* in the province of Cuneo, the highest values of prevalence, abundance and intensity belonged to the endemic species *T. retortaeformis* and *T. leporis*; (III) in *L. t. varronis*, the highest values of prevalence, abundance and intensity belonged to Cestoda tapeworms, which remained unidentified; (IV) in *S. floridanus*, the various parasites were found at higher prevalence, abundance and intensity values than in *L. europaeus* probably due to their better adaptation to their native host; and (V) in the Lagomorpha community as a whole, multi-species *Trichostrongylus* infections were a rare event. These findings indicate that, exotic parasites may be able to replace the common, native parasitofauna of *L.* europaeus in areas of sympatry with S. floridanus as the province of Alessandria. Nevertheless, where the brown hare and the eastern cottontail rabbit are not sympatric, autochthonous parasites remain prevalent even when alien parasites co-occur. Finally, the apparent exclusion of multiple infections by different *Trichostrongylus* species may be driven by a competition to occupy a similar ecological niche within the host species. A complex network of interdependent variables (e.g., host distribution range, population density, behavioural traits, diet, and immunocompetence) acts in shaping the component parasite communities of sympatric autochthonous and allochthonous terrestrial mammals [60].

The finding of *O. cuniculi*, *T. calcaratus* and *T. affinis* in an absolutely new biotic habitat, such as the gastrointestinal tract of *L. europaeus*, may in itself show how organisms are able to adapt their life history traits in order to achieve transmission. Our analysis of phenotypic plasticity, which may be a proxy for parasite fitness inside each host species, included reproductive parameters (i.e., dimensions of the reproductive apparatus in males and fertility in females) which, as suggested by many authors [16,61,62], are the ones with the highest ecological flexibility.

Our biometric measurements on *O. cuniculi*, *T. calcaratus* and *T. affinis*, and subsequent statistical tests, indicated that: (I) for *O. cuniculi*, the spicula are significantly bigger in size for individuals isolated in *L. europaeus*; (II) for *T. calcaratus*, the biometric variations of gubernaculum and spicula for male parasites, and number of eggs for females, are the widest and are significantly higher in *L. europaeus*, which may indicate a significantly higher reproductive potential than for the same parasite in *S. floridanus*; (III) for *T. affinis*, statistical analysis of the biometric measurements was very similar to that observed for *T. calcaratus* in both males and females, which further supports the adaptation of exotic parasites within a newly colonised host species.

Finally, the populations of *O. cuniculi*, *T. calcaratus* and *T. affinis* in *L. europaeus* showed higher prevalence, abundance and intensity values than in *S. floridanus*. In this context, we cannot exclude that infection levels may contribute to shaping the life history traits observed for these parasites. In fact, our data do not exclude the influence of a crowding effect. The definition of fitness is by no means simple [61] and many factors are involved. High phenotypic plasticity, with very few parasites above average, has already been established in situations where density-dependent competition takes place [63,64,65]. Another limitation of our study is that, given its limited sample size, we were not able to detect transmission of endemic parasites to the eastern cottontail rabbit. Further studies and a larger sample size would be needed to evaluate this aspect. A third limitation of our work is the absence of molecular data. Future genetic comparisons of *O. cuniculi* and *Trichostrongylus* spp. will elucidate whether the observed phenotypic plasticity signals evolutionary divergence between these parasitic species in the newly colonised *L. europaeus* and the native host *S. floridanus*.

## 5. Conclusions

The introduction of an exotic animal species, especially in the absence of adequate management, can cause serious damage to native biodiversity due to both direct competition with autochthonous animal species and parasite-mediated competition after the introduction of alien infectious agents [66,67]. An increased susceptibility of the brown hare to exotic parasites could result in its disappearance from the Piedmont territory, at least from the areas of sympatry with *S. floridanus*. This hypothesis is supported by studies demonstrating a close correlation between the extinction of an autochthonous species to the advantage of an allochthonous one due to the effects induced by exotic parasites [68]. In the UK, the competition between the native Eurasian red squirrel (*Sciurus vulgaris*) and the introduced Eastern grey squirrel (*Sciurus carolinensis* Gmelin, 1788) is known to be also mediated by a parapoxvirus, highly pathogenic in red squirrels, introduced and carried by grey squirrels, in which it has very little virulence [69,70]. The rate of red squirrel replacement by grey squirrels is twenty times faster in areas where grey squirrels carry the virus [71]. Spillover of macroparasites from the grey to the red squirrel is also documented and known to affect the survival of the native species, with a reduction of its fitness [72].

Our work represents a first exploratory study on the ecological macro-community formed by native and alien host species and their parasitofauna. Further efforts are needed to understand the strategies and factors conditioning the evolution of these parasite communities. The various hypotheses for future research are located in a field, that of parasite evolutionary ecology, which encompasses and allows the intersection of many disciplines, from human and veterinary medicine to agricultural sciences, from epidemiology to wildlife management, and whose study can help us decipher the community ecology of higher vertebrates. The initial results of our work highlight that the community of Lagomorpha in northwestern Italy represents an extremely interesting system for modelling ecological and evolutionary interactions between parasites and their hosts.

## Figures and Tables

**Table 1:** Parasite community prevalence, abundance and intensity, by host species and sampled province. Confidence intervals (CI) at 95% is provided in squared brackets.

	Species	Prevalence %	Abundance	Intensity
*Lepus europaeus* (Alessandria—N = 21)	*Obeliscoides cuniculi*	40.0 [16.8–68.7]	10.5 [−3.7–24.7]	26.2 [−13.4–26.2]
	*Trichostrongylus calcaratus*	33.3 [17.2–54.6]	27.3 [−0.4–55.0]	39 [0.9–77.1]
	*Trichostrongylus retortaeformis*	19.0 [7.7–40.0]	15.5 [−0.3–31.3]	77.5 [31.8–123.2]
	*Trichostrongylus affinis*	25.0 [11.2–46.9]	19.3 [−8.3–46.9]	77.2 [−52.7–207.1]
	*Trichuris leporis*	20.0 [8.1–41.6]	0.3 [0–0.7]	1.8 [0.3–3.2]
	*Trichostrongylus colubriformis*	4.8 [0.8–22.7]	13 [NA]	260 [NA]
*Sylvilagus floridanus* (Alessandria—N = 16)	*Obeliscoides cuniculi*	86.7 [62.1–96.3]	96.8 [−14.2–207.8]	149 [−22.6–320.6]
	*Trichostrongylus calcaratus*	85.7 [60.1–96.0]	963 [117–1809]	1123.5 [151.5–2095.5]
	*Trichostrongylus affinis*	68.8 [44.4–85.8]	79.1 [−11.6–169.8]	115 [−17.6–247.6]
*Lepus timidus varronis* (Cuneo—N = 7)	*Trichostrongylus retortaeformis*	42.9 [15.8–74.9]	1.4 [−1.0–3.8]	3.3 [−4.6–11.2]
	*Trichuris leporis*	14.3 [2.6–51.3]	0.7 [NA]	5 [NA]
	*Trichostrongylus colubriformis*	14.3 [2.6–51.3]	26.1 [NA]	183 [NA]
	Cestoda	71.4 [35.9–91.8]	91.7 [−49.7–233.1]	128.4 [−83.7–340.5]
	*Nematodirus* sp.	14.3 [2.6–51.3]	0.57 [NA]	4 [NA]
*Lepus europaeus* (Cuneo—N = 30)	*Obeliscoides cuniculi*	6.7 [1.8–21.3]	0.4 [−0.4–1.2]	6.5 [−63.6–76.6]
	*Trichostrongylus calcaratus*	3.3 [0.6–16.7]	0.2 [NA]	7 [NA]
	*Trichostrongylus retortaeformis*	40.0 [24.6–57.9]	12.3 [2.6–22.0]	30.8 [9.2–52.4]
	*Trichostrongylus affinis*	3.3 [0.6–16.7]	1 [NA]	30 [NA]
	*Trichuris leporis*	40.0 [24.6–57.9]	5.6 [1.3–9.9]	13.9 [4.5–23.3]
	*Trichostrongylus* *colubriformis*	3.3 [0.6–16.7]	14.3 [NA]	430 [NA]
	Cestoda	26.7 [14.2–44.4]	2.9 [−0.2–6.0]	10.7 [−1.1–22.5]
	*Passalurus Ambiguus*	13.3 [5.3–29.7]	26.4 [−14.4–67.0]	197.7 [–223.5–618.9]

## Data Availability

Data sharing is not applicable to this article.

## References

[1] Convention on Biological Diversity (CBD), Glossary of Terms. https://www.cbd.int/invasive/terms.shtml.

[2] McNeely J.A., McNeely J.A. (2001). The Great Reshuffling: Human Dimensions of Invasive Alien Species.

[3] Russell J.C., Towns D.R., Clout M.N. (2008). Review of rat invasion biology: Implications for island biosecurity. Sci. Conserv..

[4] Massetti M. (2008). Uomini e (Non Solo) Topi. Gli Animali Domestici e la Fauna Antropocora.

[5] Cocchi R., Volponi S., Baccetti N. (2020). Piano di Gestione Nazionale dell’Ibis Sacro Threskiornis aethiopicus (Latham, 1790).

[6] Nerva L., Iannucci A., Menchetti M., Andreoni A., Chitarra W., Martini M., Mueller N., Peeters T.M.J., Pesenti E., Verbeylen G. (2021). Where do Chip and Dale come from? Origins of invasive populations of the Siberian chipmunk in Europe. Mamm. Res..

[7] Scalera R., Bevilacqua G., Carnevali L., Genovesi P. (2018). Le Specie Esotiche Invasive: Andamenti, Impatti e Possibili Risposte.

[8] Pârâu L.G., Strubbe D., Mori E., Menchetti M., Ancillotto L., van Kleunen A., White R.L., Luna A., Hernández-Brito D., Le Louarn M. (2016). Rose-ringed Parakeet *Psittacula krameri* populations and numbers in Europe: A complete overview. Open Ornithol. J..

[9] IUCN (2018). Guidance for Interpretation of the CBD Categories of Pathways for the Introduction of Invasive Alien Species.

[10] Wauters L.A., Tosi G., Gurnell J. (2005). A review of the competitive effects of alien grey squirrels on behaviour, activity and habitat use of red squirrels in mixed, deciduous woodland in Italy. Hystrix It. J. Mamm..

[11] Zwerschke N., van Rein H., Harrod C., Reddin C., Emmerson M.C., Roberts D., O’Connor N.E. (2018). Competition between co-occurring invasive and native consumers switches between habitats. Funct. Ecol..

[12] Doherty T.S., Glen A.S., Nimmo D.G., Ritchie E.G., Dickman C.R. (2016). Invasive predators and global biodiversity loss. Proc. Natl. Acad. Sci. USA.

[13] Simberloff D. (1996). Hybridization between native and introduced wildlife species: Importance for conservation. Wildl. Biol..

[14] Largiadèr C.R. (2008). Hybridization and introgression between native and alien species. Biol. Invasions.

[15] Foster R., Peeler E., Bojko J., Clark P.F., Morritt D., Roy H.E., Stebbing P., Tidbury H.J., Wood L.E., Bass D. (2021). Pathogens co-transported with invasive non-native aquatic species: Implications for risk analysis and legislation. NeoBiota.

[16] Hudson P.J., Dobson A.P., Cattadori I.M., Newborn D., Haydon D.T., Shaw D., Benton T.G., Grenfell B.T. (2002). Trophic interactions and population growth rates: Describing patterns and identifying mechanisms. Phil. Trans. R. Soc. Lond. B.

[17] Dobson A.P., Hudson P.J. (1992). Regulation and stability of a free-living host-parasite system, *Trichostrongylus tenuis* in red grouse. II. Population models. J. Anim. Ecol..

[18] Hudson P.J., Dobson A.P., Newborn D. (1992). Do parasites make prey vulnerable to predation? Red grouse and parasites. J. Anim. Ecol..

[19] Hudson P.J., Newborn D., Dobson A.P. (1992). Regulation and stability of a free-living host-parasite system, *Trichostrongylus tenuis* in red grouse. I. Monitoring and parasite reduction experiments. J. Anim. Ecol..

[20] Hudson P.J., Dobson A.P., Newborn D. (1999). Population cycles and parasitism. Science.

[21] Newey S., Thirgood S.J., Hudson P.J. (2004). Do parasite burdens in spring influence condition and fecundity of female mountain hares *Lepus timidus*?. Wildl. Biol..

[22] Halvorsen O., Bye K. (1999). Parasites, biodiversity and population dynamics in an ecosystem in the High Arctic. Vet. Parasitol..

[23] Irvine R.J., Stien A., Halvorsen O., Langvatn R., Albon S.D. (2000). Life-history strategies and population dynamics of abomasal nematodes in Svalbard reindeer (*Rangifer tarandus platyrhynchus*). Parasitology.

[24] Daszak P., Cunningham A.A., Hyatt A.D. (2000). Emerging infectious diseases of wildlife: Threats to biodiversity and human health. Science.

[25] Kelly D.W., Paterson R.A., Townsend C.R., Poulin R., Tompkins D.M. (2009). Parasite spillback: A neglected concept in invasion ecology?. Ecology.

[26] Doria G., Cappadocia Orsini L., Doria G. (1991). Silvilago o minilepre *Sylvilagus floridanus* (Allen). Animali e Piante dalle Americhe all’Europa.

[27] Spagnesi M., Spagnesi M., De Marinis A.M. (2002). Silvilago *Sylvilagus floridanus* (J.A. Allen, 1890). Quaderni di Conservazione della Natura. Mammiferi d’Italia.

[28] Andreotti A., Baccetti N., Perfetti A., Besa M., Genovesi P., Guberti V. (2001). Mammiferi e uccelli esotici in Italia: Analisi del fenomeno, impatto sulla biodiversità e linee guida gestionali. Quaderni di Conservazione della Natura.

[29] Tizzani P., Catalano S., Rossi L., Duignan P.J., Meneguz P.G. (2014). Invasive species and their parasites: Eastern cottontail rabbit *Sylvilagus floridanus* and *Trichostrongylus affinis* (Graybill, 1924) from Northwestern Italy. Parasitol. Res..

[30] Tizzani P., Andrade D., Molinar Min A.R., Peano A., Meneguz P.G. (2020). Does the introduction of alien species represent a sanitary threat for native species? The case of the eastern cottontail *Sylvilagus floridanus* in Italy. Life.

[31] Smith R.K., Vaughan Jennings N., Harris S. (2005). A quantitative analysis of the abundance and demography of European hares *Lepus europaeus* in relation to habitat type, intensity of agriculture and climate. Mamm. Rev..

[32] Malacarne G., Cucco M., Boano G. (1999). Progetto di Piano Faunistico-Venatorio.

[33] Canova L., Gazzola A., Pollini L., Balestrieri A. (2020). Surveillance and habitat diversity affect European brown hare (*Lepus europaeus*) density in protected breeding areas. Eur. J. Wildl. Res..

[34] Naldi L., Greco I., Ferretti M., Zaccaroni M. (2020). Density estimates and habitat preferences of the European Hare *(Lepus europaeus*) on mountainous areas in Italy. Mammal Study.

[35] Silvano F., Acquarone C., Cucco M. (2000). Distribution of the eastern cottontail *Sylvilagus floridanus* in the province of Alessandria. Hystrix It. J. Mamm..

[36] Bertolino S., Ingegno B., Girardello M. (2011). Modelling the habitat requirements of invasive Eastern Cottontail (*Sylvilagus floridanus*) introduced to Italy. Eur. J. Wildl. Res..

[37] Tizzani P., Dematteis A. (2009). Linee Guida per la Gestione della Lepre Europea (Lepus europeus).

[38] Skrjabin K.I., Shikhobalova N.P., Schulz R.S. (1954). Essential of Nematodology. Trichostrongylids of Animals and Man.

[39] QGIS.org (2022). QGIS Geographic Information System. http://www.qgis.org.

[40] Tizzani P., Catalano S., Rossi L., Meneguz P.G. (2010). A new tool for the measurement of parasitic structures: A GIS approach. Parassitologia.

[41] Margolis L., Esch G.W., Holmes J.C., Kuris A.M., Schad G.A. (1982). The use of ecological terms in parasitology (report of an ad hoc committee of the American Society of Parasitologists). J. Parasitol..

[42] Bush A.O., Lafferty K.D., Lotz J.M., Shostak A.W. (1997). Parasitology meets ecology on its own terms: Margolis et al. revisited. J. Parasitol..

[43] R Core Team R: A Language and Environment for Statistical Computing. https://www.R-project.org/.

[44] Kassambara A. (2020). Ggpubr: ‘Ggplot2′ Based Publication Ready Plots. https://CRAN.R-project.org/package=ggpubr.

[45] Chen H. (2021). VennDiagram: Generate High-Resolution Venn and Euler Plots. https://CRAN.R-project.org/package=VennDiagram.

[46] Smith K.F., Sax D.F., Lafferty K.D. (2006). Evidence for the role of infectious disease in species extinction and endangerment. Conserv. Biol..

[47] Delogu M., Ghetti G., Gugiatti A., Cotti C., Piredda I., Frasnelli M., De Marco M.A. (2013). Virological investigation of avian influenza virus on postglacial species of Phasianidae and Tetraonidae in the Italian Alps. Int. Sch. Res. Not..

[48] Sinclair R., Melville L., Sargison F., Kenyon F., Nussey D., Watt K., Sargison K. (2016). Gastrointestinal nematode species diversity in Soay sheep kept in a natural environment without active parasite control. Vet. Parasitol..

[49] Leivesley J.A., Bussière L.F., Pemberton J.M., Pilkington J.G., Wilson K., Hayward A.D. (2019). Survival costs of reproduction are mediated by parasite infection in wild Soay sheep. Ecol. Lett..

[50] Iacopelli F., Fanelli A., Tizzani P., Berriatua E., Prieto P., Martínez-Carrasco C., León L., Rossi L., Candela M.G. (2020). Spatio-temporal patterns of sarcoptic mange in red deer and Iberian ibex in a multi-host natural park. Res. Vet. Sci..

[51] Carrau T., Martínez-Carrasco C., Garijo M.M., Alonso F., de Ybáñez R.R., Tizzani P. (2021). Multivariate abundance analysis of multi-host/multi-parasite lungworms in a sympatric wild ruminant population. Diversity.

[52] Carrau T., Martínez-Carrasco C., Garijo M.M., Alonso F., Vizcaíno L.L., Herrera-Russert J., Tizzani P., de Ybáñez R.R. (2021). Epidemiological approach to nematode polyparasitism occurring in a sympatric wild ruminant multi-host scenario. J. Helminthol..

[53] Fanelli A., Menardi G., Chiodo M., Giordano O., Ficetto G., Bessone M., Lasagna A., Carpignano M.G., Min A.M., Gugiatti A. (2020). Gastroenteric parasite of wild Galliformes in the Italian Alps: Implication for conservation management. Parasitology.

[54] Tizzani P., Menzano A., Catalano S., Rossi L., Meneguz P.G. (2011). First report of *Obeliscoides cuniculi* in European brown hare (*Lepus europaeus*). Parasitol. Res..

[55] Alzaga V., Tizzani P., Acevedo P., Ruiz-Fons F., Vicente J., Gortázar C. (2009). Deviance partitioning of host factors affecting parasitization in the European brown hare (*Lepus europaeus*). Naturwissenschaften.

[56] Gontero C., Fanelli A., Zanet S., Meneguz P.G., Tizzani P. (2020). Exotic species and autochthonous parasites: *Trichostrongylus retortaeformis* in Eastern cottontail. Life.

[57] Soveri T., Valtonern M. (1983). Endoparasites of hares (*Lepus timidus* L. and *L. europaeus* Pallas) in Finland. J. Wild. Dis..

[58] Bordes F., Langand J., Feliu C., Morand S. (2007). Helminth communities of an introduced hare (*Lepus granatensis*) and a native hare (*Lepus europaeus*) in southern France. J. Wild. Dis..

[59] Torchin M.E., Lafferty K.D., Dobson A.P., McKenzie V.J., Kuris A.M. (2003). Introduced species and their missing parasites. Nature.

[60] Kołodziej-Sobocińska M. (2019). Factors affecting the spread of parasites in populations of wild European terrestrial mammals. Mamm. Res..

[61] Stearns S.C. (1992). The Evolution of Life Histories.

[62] Poulin R. (2007). Evolutionary Ecology of Parasites.

[63] Keymer A.E. (1982). Density-dependent mechanisms in the regulation of the intestinal helminth populations. Parasitology.

[64] Shostak A.W., Dick T.A. (1987). Individual variability in reproductive success of *Triaenophorus crassus* Forel (Cestoda: Pseudophyllidea), with comments on the use of the Lorenz curve and Gini coefficient. Can. J. Zool..

[65] Szalai A.J., Dick T.A. (1989). Differences in numbers and inequalities in mass and fecundity during the egg-producing period for *Raphidascaris acus* (Nematoda: Anisakidae). Parasitology.

[66] Dove A.D.M. (2000). Richness patterns in the parasite communities of exotic poeciliid fishes. Parasitology.

[67] Rizzo D.M., Garbelotto M. (2003). Sudden oak death: Endangering California and Oregon forest ecosystems. Front. Ecol. Environ..

[68] De Castro F., Bolker B. (2005). Mechanisms of disease-induced extinction. Ecol. Lett..

[69] Tompkins D.M., Sainsbury A.W., Nettleton P., Buxton D., Gurnell J. (2002). Parapoxvirus causes a deleterious disease in red squirrels associated with UK population declines. Proc. Biol. Sci..

[70] Tompkins D.M., White A.R., Boots M. (2003). Ecological replacement of native red squirrels by invasive greys driven by disease. Ecol. Lett..

[71] Rushton S.P., Lurz P.W.W., Gurnell J., Nettleton P., Bruemmer C., Shirley M.D.F., Sainsbury A.W. (2006). Disease threats posed by alien species: The role of a poxvirus in the decline of the native red squirrel in Britain. Epidemiol. Infect..

[72] Romeo C., Piscitelli A.P., Santicchia F., Martinoli A., Ferrari N., Wauters L.A. (2021). Invading parasites: Spillover of an alien nematode reduces survival in a native species. Biol. Invasions.

